# RF-EMF Exposure Assessment: Comparison of Measurements in Airports and Flights with and Without Wi-Fi Service

**DOI:** 10.3390/s25216710

**Published:** 2025-11-03

**Authors:** Enrique Arribas, Isabel Escobar, Antonio Martinez-Plaza, Montaña Rufo-Pérez, Antonio Jimenez-Barco, Jesús M. Paniagua-Sánchez, Pilar Marín, Raquel Ramirez-Vazquez

**Affiliations:** 1 Department of Physics, Faculty of Computer Science Engineering, University of Castilla-La Mancha, Avda. de España s/n, University Campus, 02071 Albacete, Spain; enrique.arribas@uclm.es (E.A.); isabelmaria.escobar@uclm.es (I.E.); 2 MORFEO Research Group, University of Castilla-La Mancha, 02071 Albacete, Spain; antonio.mplaza@uclm.es; 3 Mathematics Department, School of Industrial Engineering, University of Castilla-La Mancha, Avda. de España s/n, University Campus, 02071 Albacete, Spain; 4 Applied Physics Department, Polytechnic School, University of Extremadura, Avda. de la Universidad s/n, 10003 Cáceres, Spain; mmrufo@unex.es (M.R.-P.); ajimenez@unex.es (A.J.-B.); paniagua@unex.es (J.M.P.-S.); 5 Instituto de Magnetismo Aplicado (IMA), Universidad Complutense de Madrid (UCM-ADIF-CSIC), 28230 Las Rozas, Spain; mpmarin@fis.ucm.es; 6 Departamento de Física de Materiales, Universidad Complutense de Madrid (UCM), 28040 Madrid, Spain; 7 Department of Physics, Polytechnic School of Cuenca, University of Castilla-La Mancha, University Campus, 16071 Cuenca, Spain; 8 ESAT-WaveCoRE, Department of Electrical Engineering, Katholieke Universiteit Leuven, Kasteelpark Arenberg 10, Box 2444, 3001 Leuven, Belgium

**Keywords:** personal exposure, Radiofrequency Electromagnetic Fields, airplane with and without Wi-Fi connection, intensity of electromagnetic wave

## Abstract

**Highlights:**

**What is the main findings?**

- Personal exposure to RF-EMF from Wi-Fi on four international flights.

**What is the implication of the main finding?**

- Measurement inside an airplane with and without Wi-Fi connection using personal exposimeters.

**Abstract:**

This paper presents the results of personal exposure measurements to Radiofrequency Electromagnetic Fields from 2.4 GHz and 5.85 GHz Wi-Fi frequency bands. Measurements were taken in several specific scenarios: within international airports terminals, during takeoff, inside airplanes while flying with and without onboard Wi-Fi service (including while actively using a Wi-Fi connection), and during landing. Data were recorded onboard four international flights (two-round trip flights), from Spain to Mexico, and from Spain to Belgium. Two personal exposimeters, EME SPY 140 and EME Spy Evolution, were used to collect intensity level measurements in each scenario. During the outbound, the mean exposure value inside the airplane flight was 93.9 µW/m^2^ in the 2.4 GHz Wi-Fi frequency band and 46.4 µW/m^2^ in the 5.85 GHz Wi-Fi band (Spain to Mexico), and 7.29 µW/m^2^ in the 2.4 GHz Wi-Fi band and 2.40 µW/m^2^ in the 5.85 GHz Wi-Fi band (Spain to Belgium). For the return flight, the average value was 26.7 µW/m^2^ in the 2.4 GHz Wi-Fi band and an average of 9.87 µW/m^2^ in the 5.85 GHz Wi-Fi band (Mexico to Spain), and 3.24 µW/m^2^ in the 2.4 GHz Wi-Fi band and 1.23 µW/m^2^ in the 5.85 GHz Wi-Fi band (Belgium to Spain). Personal exposure levels to RF-EMFs from the Wi-Fi frequency band inside an airplane, even at the airport, are very low and well below the reference levels established by the international guidelines (10 W/m^2^).

## 1. Introduction

Measuring personal exposure to Radiofrequency Electromagnetic Fields (RF-EMFs) enables the determination of intensity levels in various microenvironments. The implementation of new methodologies for measuring electromagnetic wave intensity provides insight into personal exposure levels to RF-EMFs for the general public, in sensitive centers (such as schools, nurseries, and hospitals, etc.), and in different scenarios (microenvironments) under study in which RF-EMF intensity levels have been measured and simulated.

These types of studies have been carried out using various methodologies and different means of transport, as well as measuring equipment [1,2,3,4,5,6,7,8,9,10,11,12,13,14,15,16,17,18,19,20,21]. For example, carrying the personal exposimeter in a cloth bag on a researcher’s or a volunteer’s body [14,15], placing the personal exposimeter in a plastic basket in front of a bicycle, placing the exposimeter in a plastic or cardboard-made tube carried by a researcher [22,23,24], an exposimeter mounted on a car rooftop, or mounting six ExpoM-RF devices in a polystyrene box inside a wooden handcart [25], also including experiments with a drone [26,27]. Measurements have also been made inside an airplane [28]. This last study was carried out by the authors of the present work, and a very reduced version of RF-EMF exposure measurements from 2 GHz Wi-Fi and 5 GHz Wi-Fi frequency bands on a regular flight from Barajas Adolfo Suarez Airport in Madrid (Spain) to Benito Juárez International Airport in Mexico City (Mexico), outbound flight, was presented at the ICEMS-BIOMED 2022 conference [28], in order to publicize the previous results of what the research group was conducting.

The majority of studies on personal RF-EMF exposure have been conducted with volunteer participants, with measurements differentiated by frequency bands and categorized according to the various microenvironments assessed [1,2,3,6,9,13,14,15,16,19,20,21,29,30,31,32]; most of them were carried out in European countries [1,2,3,4,6,7,9,11,13,14,15,16,18,19,20,21,22,26,29,30,32,33,34,35,36,37] and a first study on RF-EMF exposure measurements was conducted in a Mexican city [15].

The growing trend in wireless devices and Wi-Fi networks poses significant management challenges to network administrators [38] and society in general. The increasing quality brought in by Wi-Fi technology makes means of transport more advantageous in enclosed and controlled environments such as a cabin airplane [28,39], and even in a train, bus, or car [40].

Airplanes have always been vehicles in which the use of electronic devices is forbidden during flights to avoid interference with the aircrew’s radio, which is an indispensable element for communication between pilots and control towers. Fixed mobile phone towers establishing high-speed internet connectivity cannot be installed and connected everywhere because of the presence of oceans and seas between countries, and the solution is to use space to connect two points worldwide [41], which involves the use of satellite connectivity as in airplanes. In the civil market, Unmanned Aerial Vehicle (UAV) systems most often use frequencies close to Wi-Fi [42].

To achieve Wi-Fi connectivity on airplanes, it is not enough to place an antenna outside the airplane; the airplane also needs to be prepared [43]. Wi-Fi access points are enabled so that passengers can be connected from their mobile, tablet, or laptop, as would happen with a conventional router to enjoy the Wi-Fi service, demanded by customers to connect to the internet.

Connectivity patterns can be easily disrupted by commercially available devices that interfere with Wi-Fi signals. Expanding communication capabilities between the operator and the aircraft involves access to a broader range of radio frequencies, which in turn supports the adoption of technologies with greater resistance to interference [42]. Onboard Wi-Fi connections operate at cruising altitudes above 10000 m, with devices set to airplane mode. In the case of Iberia, the airline where this study was conducted, a satellite-based global coverage service is provided. The speed of the connection varies depending on the type of device in use and the number of users connected simultaneously [44].

Coupled with the demand for Wi-Fi service inside an airplane and the need to be connected to attend to personal and work issues, especially on very long trips (as in this case), there is the concern of the general population about possible negative effects on the health of these RF-EMFs. In this context, few studies have addressed this issue and to the best of our knowledge, there are no results from studies focused on the Wi-Fi band inside an airplane that have measured personal exposure from this band at different times of the flight.

Michałowska has assessed the electromagnetic fields produced by training aircraft and shows that the results do not exceed the allowable reference levels [45,46,47]. In a recent study, measurements were carried out on three commercial flights, confirming the current negligible values compared with the established regulatory limits. In this case, the main important measured levels before takeoff were UL (Upload) cellular frequency bands, with a maximum of 0.018 W/m^2^ in the 2.6 GHz frequency band, and after landing the higher power density level was 0.031 W/m^2^ (in the UL cellular frequency band) in 1.9 GHz [48].

The present study aimed to measure personal exposure to RF-EMFs from the 2.4 to 2.5 GHz Wi-Fi band (referred to as 2.4 GHz) and 5.15 to 5.85 GHz Wi-Fi band (referred to as 5.85 GHz) inside an airplane while flying with and without Wi-Fi service, on board four international flights (two round trips); the first from Spain to Mexico and the second from Spain to Belgium. The decision to measure the Wi-Fi band inside an airplane was motivated by the publication of an article by Pall regarding Wi-Fi as an important threat to human health [49] which has received scientific commentaries [50].

Another motivation for carrying out and writing this article has been based on the results obtained on a flight from Madrid to Mexico City (CDMX) with a Wi-Fi connection [28]. This first study only presented results from a single flight, and upon seeing the results it was decided to compare the results from the outbound flight (Madrid to Mexico City) with the results from the return flight (Mexico City to Spain), and also a flight using Wi-Fi. In addition to the second return flight (Mexico City to Madrid), measurements were taken on two return flights (Spain to Belgium and Belgium to Madrid). This flight was shorter, lasting 2.5 h compared to the 13.5 h flight, and included a flight that did not offer Wi-Fi service to its passengers. This second flight allowed researchers to compare RF-EMF exposure measurements on an airplane without Wi-Fi service with those taken on an airplane that did offer Wi-Fi service. These measurements, as it turns out, are the ones discussed in this publication.

Measurements of RF-EMFs inside buildings, including on public roads and land transportation, are already being conducted, but there is little precedent for such measurements inside airplanes.

## 2. Materials and Methods

### 2.1. Devices and Studio Microenvironment

Measurements of personal exposure to RF-EMFs were carried out with two personal exposimeters, EME SPY 140 and EME Spy Evolution, for both flights. The first exposimeter measures 14 frequency bands ranging from 88 MHz to 5.85 GHz, while the second exposimeter can monitor up to 20 user-defined frequency bands selected from 84 bands preconfigured by the Microwave Vision Group (MVG Industries, Villejust, France), covering the range from 80 MHz to 6 GHz [51,52].

Exposimeters are band-selective, meaning they measure the bands separately. Both devices can distinguish the contributions from each emitting frequency band and the total field. For this study, focus was placed on the two Wi-Fi frequency bands, 2.4 GHz (2.4–2.5 GHz) and 5.85 GHz (5.15–5.85 GHz). The detection limits of the two devices for the two bands are 0.005 and 0.02 V/m for the Spy 140 in 2.4 GHz and 5.85 GHz Wi-Fi, respectively, and 0.02 and 0.05 V/m for the Spy Evolution in 2.4 GHz and 5.85 GHz Wi-Fi, respectively.

Measurements were conducted by the researcher, inside an airplane during four direct international flights. The first trip was from the Adolfo Suárez Madrid-Barajas Airport (Spain) to Benito Juárez International Airport (Mexico), while the second trip was from the Adolfo Suárez Madrid-Barajas Airport (Spain) to Brussels Zaventem (Belgium).

The first two flights of the first trip lasted approximately 11 h each, and the last two flights of the second trip lasted approximately 2 h each. Measurements were taken at airport terminals and onboard four commercial flights. Inside the cockpit, the researcher sat in the center section of the plane. Throughout the flights, the researcher carried out the personal exposimeter in a cloth bag, hanging on the side near the waist (one on the right side and the other on the left side). The personal mobile phone was kept in airplane mode and away from the exposimeters (approximately 1 m), except for a few minutes before and after it was used to communicate using the Wi-Fi connection, these activities were recorded in the researcher’s diary notebook during the first trip.

The recording interval was 10 s. During the four flights with Wi-Fi onboard, connectivity was maintained in the background (session started); brief periods of active mobile phone use (such as messaging and downloading) occurred at predefined times to simulate typical passenger behavior. The measurements were carried out during for the entire duration of both trips, approximately 13.5 h and 2.5 h, respectively, that is, they were continuous measures. Also, at different times when the phone was connected to the Wi-Fi network, the use was continuous and uninterrupted (messaging or downloading).

Several measurement scenarios were considered as different microenvironments: (1) inside the airport and at the moment of boarding the airplane, (2) inside the airplane (using Wi-Fi while waiting to take off in the country of origin and disembarkation in the country of destination), (3) at the moment of taking off and landing (devices in flight mode), and (4) inside the airplane during flight (using and not using the Wi-Fi service provided by the airline).

During the first trip (Spain to Mexico and Mexico to Spain), the researcher paid a Wi-Fi connection fee (a service provided by the airline to passengers) to use the internet and identify differences in personal exposure to RF-EMF measurements while connected and disconnected to the Wi-Fi. The Wi-Fi service contract took place at three different times during each flight (three times for each flight), with each contract lasting 1 h, and the fee allowed the researcher to chat, search for information on the internet, receive, download, and view files with information, pictures, and short videos.

During the second trip (Spain to Belgium and Belgium to Madrid) (both flights), the researcher did not pay any fee as the airline did not provide Wi-Fi service to the passengers. This allowed for a comparison of results between flights with and without Wi-Fi service.

In the case of the first trip, measurements were carried out using a Wi-Fi connection, during which contact was established via WhatsApp with another member of the research group. An information file, pictures, and short videos were sent by this member. Internet searches were also conducted to increase activity while connected to the Wi-Fi service. These activities took place during both flights. To monitor and synchronize events for this planned activity, a WhatsApp message was sent to the researcher in Spain, who responded with information during the flight (when connected to Wi-Fi) at the time of boarding and upon payment of the fee to access the Wi-Fi connection. In the case of the second trip, measurements were performed continuously throughout the flight without any interruption, as Wi-Fi service was not available.

The EME SPY 140 and EME SPY Evolution personal exposimeters were programmed to measure every 10 s. Firstly, it was verified that the measurements of both exposimeters were comparable to avoiding differences between the devices. Recorded values below the Detection limit from the exposimeters are called “nondetects”. Subsequently, the Nondetected Data (ND) was identified, and treatment was given. The ND percentage range was approximately 75% for these two bands and for each flight. Nondetect values were replaced by the detection limit divided by two [53].

### 2.2. Statistical Analysis

The results from both devices were very similar, with no significant differences. Therefore, the measurements from both devices were average. The data were then categorized based on the microenvironment, taking into account the notes recorded in the personal diary. The average of 6 min for local exposure and the average of 30 min for whole-body exposure were calculated [54] for the different measured microenvironments.

Statistical analyses were carried out using the personal exposimeter software, EME SPY Analysis V3 and EME SPY Evolution Analysis, Statistical Package for the Social Sciences v24 (SPSS software) and Microsoft Excel 365 files were used for data processing and descriptive analyses; Google Earth Pro 7.3.6.10441 (64-bit), Iberia App, the Ryanair App, and Flightradar 24 Flight Tracker App were the applications used for flight tracking. On both flights, outbound and return (Madrid–Mexico, and Madrid–Brussels) the same aircraft model was used for all measurements on a given trip, and the flight was direct without interruptions.

Results for the intensity of the electromagnetic wave were expressed in µW/m^2^ with three significant figures [55]. Values below the detection limit, called “nondetects”, were recorded from the exposimeters in the two frequency bands for 2.4 GHz Wi-Fi and 5.85 GHz Wi-Fi.

## 3. Results

After conducting statistical analyses and reviewing the intensity levels of the electromagnetic waves, also referred to as wave intensity or power flux density (equivalent terms), for both the outbound and return flights, the highest average values were detected at the airports in Madrid (Adolfo Suárez Barajas) and CDMX (Benito Juárez) during the first trip. For the second trip, the maximum average values were measured inside the airplane while waiting for takeoff in Madrid and during disembarkation in Brussels.

**Figure 1:**
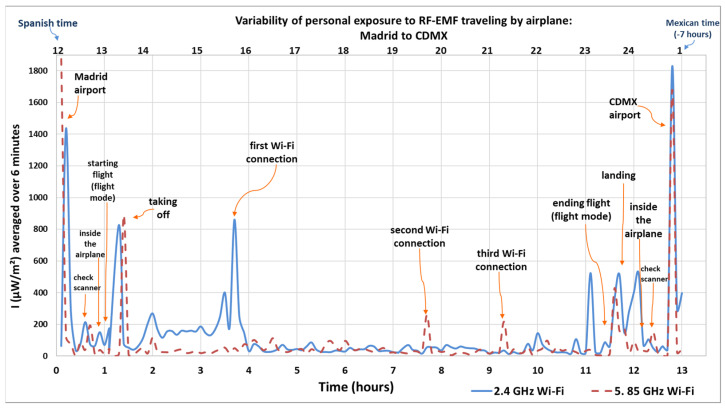
Personal exposure to RF-EMFs when traveling by airplane from Madrid to CDMX (outbound flight). Intensity of electromagnetic wave in µW/m^2^, average over 6 min. Own elaboration based on [28].

**Figure 2:**
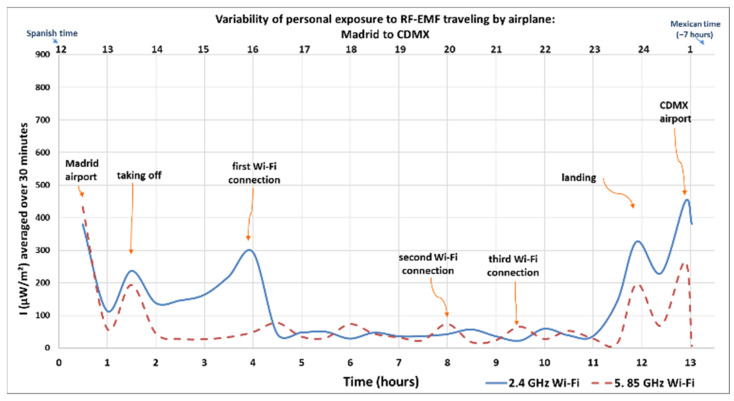
Variability of personal exposure to RF-EMFs when traveling by airplane from Madrid to CDMX (outbound flight). Intensity of electromagnetic wave in µW/m^2^, average over 30 min. Own elaboration based on [28].

**Figure 3:**
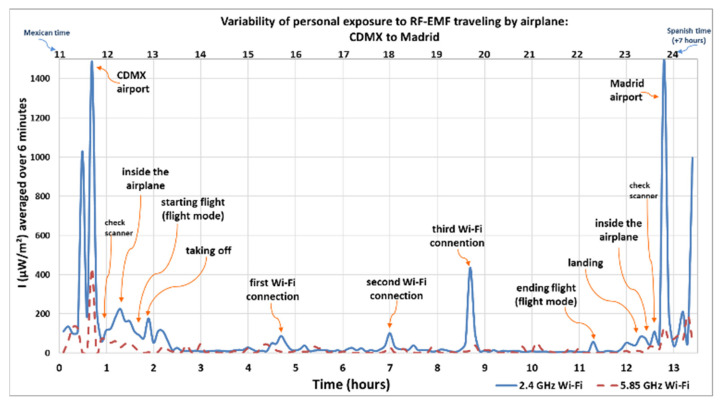
Variability of personal exposure to RF-EMFs when traveling by airplane from CDMX to Madrid (return flight). Intensity of electromagnetic wave in µW/m^2^, average over 6 min.

**Figure 4:**
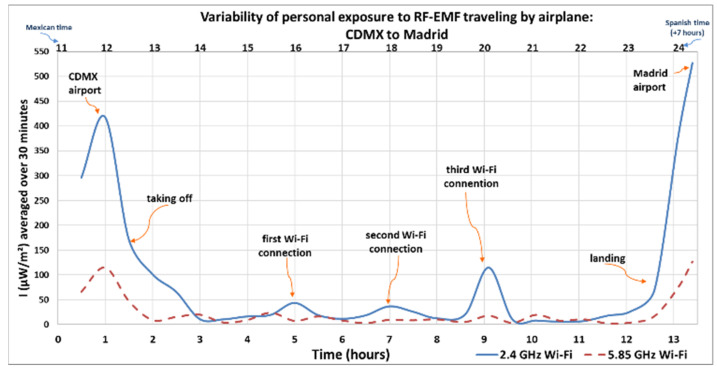
Variability of personal exposure to RF-EMFs when traveling by airplane from CDMX to Madrid (return flight). Intensity of electromagnetic wave in µW/m^2^, average over 30 min.

Figure 1, Figure 2, Figure 3 and Figure 4 show how intensity values change when the researcher was inside the airplane, with devices in flight mode, and during the flight (using and not using Wi-Fi). Another maximum average value was recorded when the researcher walked near the security check scanner at the airport, scanned separately by a device scanner (tunnel), during the first round trip from Spain to Mexico.

The Y-axis in Figure 1 and Figure 2 represents the intensity of the electromagnetic waves (µW/m^2^), averaged over 6 and 30 min, respectively, reflecting local exposure [54] from the 2.4 GHz and 5.85 GHz Wi-Fi frequency bands during both the outbound and return flights. The lower X-axis shows the elapsed time in hours, starting from when the researcher entered the airport in the departure country (marked as hour zero) until arrival at the destination. Meanwhile, the upper X-axis indicates the actual time at which each measurement was taken. The personal exposimeters were set to the time zone for the country of origin and the time zone was not modified between regions. The arrows indicate the microenvironments, or the activity recorded in the personal diary, highlighting peaks and maximum values.

Upon the departure and arrival of the flights, maximum values were recorded. Attempts were made to obtain information from the airlines at those specific moments; however, it was reported that this information could not be shared due to confidentiality reasons, and it was not possible to obtain any details on Wi-Fi operation inside the airplane.

Another maximum value was recorded during the use of Wi-Fi by the researcher who carried out the measurements, paid for the Wi-Fi service and used it to chat, search information on the internet, download information files, and view pictures and short videos received from another research group member (scheduled activity). This event occurred during both the outbound flight and the return flight.

The maximum value recorded during the entire outbound flight, including being at the airport (Figure 1) was 1870 µW/m^2^ in the 5.85 GHz Wi-Fi band, and 1830 µW/m^2^ in the 2.4 GHz Wi-Fi, inside the airport in Madrid and in CDMX, respectively. The other maximum value recorded was taking off with 827 µW/m^2^ in the 2.4 GHz Wi-Fi frequency band and 889 µW/m^2^ in the 5.85 GHz Wi-Fi frequency band (origin country), and landing was 521 µW/m^2^ in the 2.4 GHz Wi-Fi frequency band (destination country). The lowest recorded values were 12.9 µW/m^2^ in the 2.4 GHz Wi-Fi band and 4.16 µW/m^2^ in the 5.85 GHz Wi-Fi band. The 2.4 GHz band showed higher activity compared to the 5.85 GHz band.

Additionally, during the flight from Madrid to CDMX, peaks in radiation levels were recorded during three separate instances of Wi-Fi usage inside the airplane, with values reaching 861 µW/m^2^ in the 2.4 GHz Wi-Fi band and 102 µW/m^2^ in the 5.85 GHz Wi-Fi band during first Wi-Fi connection, 3.5 h after departure from the Iberian Peninsula; 69.7 µW/m^2^ in the 2.4 GHz Wi-Fi band and 261 µW/m^2^ in the 5.85 GHz Wi-Fi band during the second Wi-Fi connection, 7.5 h after departure; and 144 µW/m^2^ in the 2.4 GHz Wi-Fi band and 217 µW/m^2^ in the 5.85 GHz Wi-Fi band during the third Wi-Fi connection, and 9.5 h after the measurement process.

Figure 2 shows the variation in average intensity levels over 30 min for whole-body exposure during the outbound flight [54]. The maximum recorded values occurred when the researcher was at the origin and destination airports, in Madrid and in CDMX, were 380 µW/m^2^ in the 2.4 GHz Wi-Fi band and 433 µW/m^2^ in the 5.85 GHz Wi-Fi band, respectively. The maximum recorded values when the flight departed were 237 µW/m^2^ in the 2.4 GHz Wi-Fi band and 327 µW/m^2^ in the 2.4 GHz Wi-Fi band when the flight arrived. During the moments when Wi-Fi was accessed and used inside the airplane, values were 295 µW/m^2^ in the 2.4 GHz Wi-Fi band (first Wi-Fi connection), 74.1 µW/m^2^ in the 5.85 GHz Wi-Fi band (second Wi-Fi connection), and 65.9 µW/m^2^ in the 5.85 GHz Wi-Fi band (third Wi-Fi connection).

Figure 3 shows the average intensity levels over 6 min measured during the return flight, from Mexico to Spain, over 6 min for local exposure [54]. The Y-axis presents intensity values from the 2.4 GHz Wi-Fi and 5.85 GHz Wi-Fi bands, while the lower X-axis represents time in hours, starting from the moment that the researcher entered the airport in the country of origin until arrival in the country of destination. The upper X-axis indicates the actual time at which each measurement was recorded. The measurements follow a similar pattern to those recorded during the outbound flight.

On the return flight, from Mexico to Spain, the exposure levels are slightly lower compared to the outbound flight, this being because the airplane was smaller and fewer passengers were traveling. The Wi-Fi intensity was also less powerful, and there were more interruptions when connecting.

During the return flight, the maximum values were recorded when the researcher was at the airport of the origin country (CDMX), at the airport of the destination country (Spain), and when the airplane was taking off and landing. At the three different moments during the flight, the researcher paid for and used the Wi-Fi connection to chat, search for information on the internet, receive, download, and view files with information, pictures, and short videos sent to her by another researcher from the same group, as agreed on previously.

The maximum average values recorded during the return flight, including time spent at the airports (Figure 3 in CDMX and Madrid), were 1490 µW/m^2^ in the 2.4 GHz Wi-Fi frequency band and 1500 µW/m^2^ in the 2.4 GHz Wi-Fi frequency band, respectively. The minimum value was 3.44 µW/m^2^ in the 2.4 GHz Wi-Fi band and 1.06 µW/m^2^ in the 5.85 GHz Wi-Fi band. During this flight, the most active frequency band was 2.4 GHz Wi-Fi compared to the 5.85 GHz Wi-Fi band, like the outbound flight. When the flight was taking off, a maximum value was recorded with 177 µW/m^2^ in the 2.4 GHz Wi-Fi frequency band, and 111 µW/m^2^ in the 2.4 GHz Wi-Fi band when the airplane landed.

Figure 4 shows the variation in average intensity levels recorded during the return flight, over 30 min for whole body exposure [54]. Maximum values were measured at the airports, in CDMX and Madrid, and were recorded at 418 µW/m^2^ in 2.4 GHz Wi-Fi band and 528 µW/m^2^ in the 2.4 GHz Wi-Fi band, respectively. When the flight departed, the maximum value was 172 µW/m^2^ in the 2.4 GHz Wi-Fi band and when the flight arrived, the value was 70.5 µW/m^2^ in 2.4 the GHz Wi-Fi band. At different connection moments and use of the Wi-Fi provided by the airline, values were 43.8 µW/m^2^ in the 2.4 GHz Wi-Fi band (first Wi-Fi connection), 36.9 µW/m^2^ in the 2.4 GHz Wi-Fi band (second Wi-Fi connection), and 115 µW/m^2^ in the 2.4 GHz Wi-Fi band (third Wi-Fi connection).

**Figure 5:**
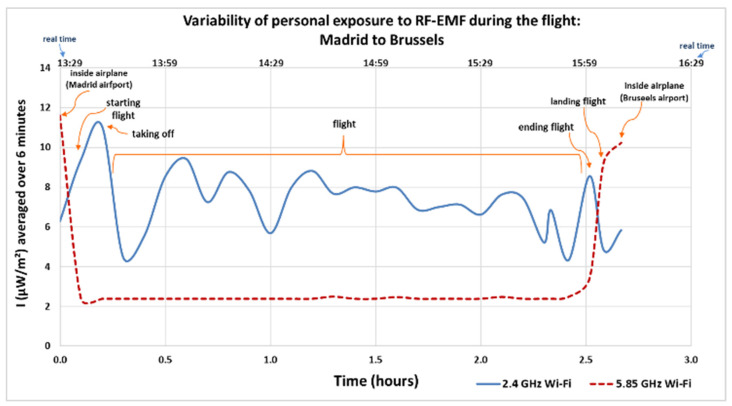
Variability of personal exposure to RF-EMFs when traveling by airplane from Madrid, Spain to Brussels, Belgium (outbound flight). Intensity of electromagnetic wave in µW/m^2^, average over 6 min.

**Figure 6:**
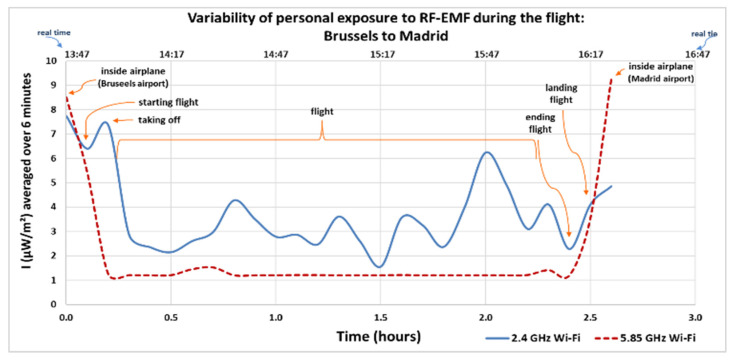
Variability of personal exposure to RF-EMFs when traveling by airplane from Brussels, Belgium to Madrid, Spain (return flight). Intensity of electromagnetic wave in µW/m^2^, average over 6 min.

Figure 5 and Figure 6 show the electromagnetic wave intensity values (µW/m^2^) recorded and averaged over a 6 min period for local exposure [54], in the 2.4 GHz and 5.85 GHz Wi-Fi bands during the outbound and return flights of the second trip from Spain to Belgium. The lower X-axis represents the elapsed time in hours, starting at the moment the researcher boarded the airplane in the country of origin (zero hours) until arrival in the destination country. The upper X-axis indicates the actual local time at which each measurement was taken.

The maximum average values recorded during the outbound flight (Figure 5), from the time the researcher boarded the airplane in the original country until the disembarkation began in the destination country, were recorded at the two airports inside the airplane, both at origin and arrival (Madrid and Brussels). The maximum values were 11.6 µW/m^2^ in the 5.85 GHz Wi-Fi frequency band (origin country) and 10.2 µW/m^2^ in the 5.85 GHz Wi-Fi frequency band, respectively (destination country). The minimum value was 2.39 µW/m^2^ in the 5.85 GHz Wi-Fi band.

During the return flight (Figure 6), the highest average values were recorded inside the airplane in the countries of origin and arrival (Brussels and Madrid), at 8.50 µW/m^2^ and 9.28 µW/m^2^, respectively, both in the 5.85 GHz Wi-Fi band. The minimum recorded value was 1.19 µW/m^2^ in the same band.

**Figure 7:**
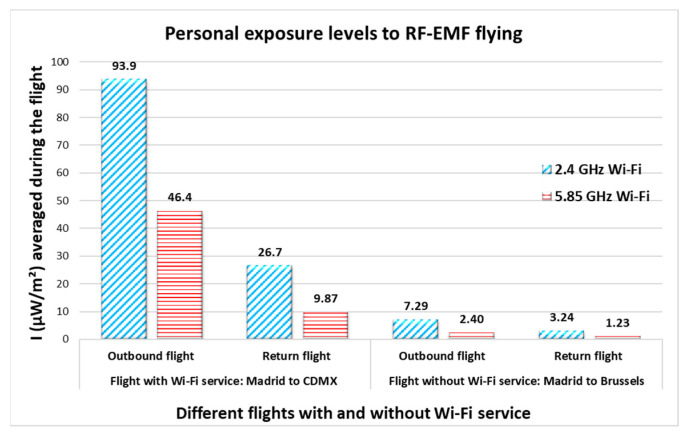
Personal exposure to RF-EMF from Wi-Fi inside an airplane flying: Madrid, Spain to CDMX, Mexico and Madrid, Spain to Brussels, Belgium, round trip, respectively. Flights with and without Wi-Fi (the intensity of electromagnetic wave in µW/m^2^).

Up to this point, only the maximum values recorded during the entire trip have been discussed. The following section presents and discusses the average values for measurements recorded in each microenvironment. Table 1 and Table 2 present the total average for personal exposure levels to RF-EMFs recorded at different times and in the various microenvironments present during the outbound flight and return flights of the first and second trips. The values measured during the flights are highlighted in Figure 7.

For the first trip, the maximum averages were inside the airports with 343 µW/m^2^ in the 5.85 GHz Wi-Fi band in Madrid and 442 µW/m^2^ in the 2.4 GHz Wi-Fi band in CDMX, for the outbound flight. Additionally, 451 µW/m^2^ inside the airport in CDMX and 445 µW/m^2^ inside the airport in Madrid, both in the 2.4 GHz Wi-Fi band.

On the second trip, the maximum averages were inside the airplane (origin and destination country) with 11.6 µW/m^2^ inside the airplane in Madrid and 10.2 µW/m^2^ inside the airplane in Brussels, both in the 5.85 GHz Wi-Fi band, for the outbound flight. And 8.50 µW/m^2^ inside the airplane in Brussels and 9.28 µW/m^2^ inside the airplane in Madrid, both in the 2.4 GHz Wi-Fi band, for the return flight.

**Figure 8:**
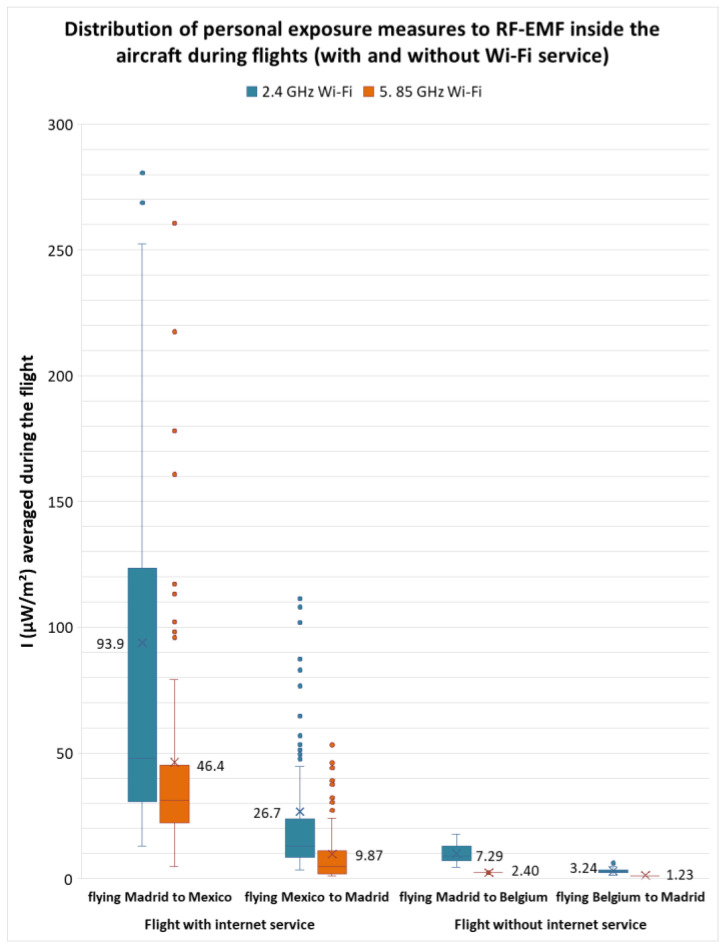
Distribution of personal exposure to RF-EMF inside the aircraft during flights (with and without Wi-Fi service): Madrid, Spain to CDMX, Mexico and Madrid, Spain to Brussels, Belgium, round trip, respectively.

The average intensity levels measured inside the airplane during the first and second trips differed. This is because the airline provided Wi-Fi service during the first trip but not during the second trip (Figure 7 and Figure 8).

Figure 8 shows the distribution of the measurements recorded during the different flights, with and without Wi-Fi service, for each frequency band. The values shown in the box plot are the average values obtained during the flight, which coincides with those in Figure 7.

The average values measured inside the airplane during the flight are comparable to the results provided by other studies measuring personal exposure to RF-EMF in other microenvironments across Wi-Fi frequency bands, in which 2.4 GHz and 5.85 GHz Wi-Fi frequency bands were subject to measurement (Table 3).

The highest levels near boarding/disembarking, that is, at the departure and destination airports, coincide with the aircraft’s takeoff and landing. This is because in both places there is much greater Wi-Fi usage activity because there are many people using the Wi-Fi connection with their mobile phones, tablets, or computers. This activity decreases shortly before takeoff and increases again upon landing. High values were also identified when using a mobile phone connected to the Wi-Fi network on the plane; the connection onboard usually occurs after logging in after purchasing the data plan and this process involves synchronizing and transferring numerous data items.

## 4. Discussion

When comparing the intensity levels measured in the different microenvironments with those measured inside the airplane during the flight, the maximum value was 500 µW/m^2^, recorded in a study carried out in Mexico with volunteer participants [15]. It should also be noted that, in Section 3, a maximum value was reported inside the airports, for example, 1870 µW/m^2^ in the 5.85 GHz Wi-Fi band (Madrid airport) and maximum values were also recorded when the plane took off, for example, 889 µW/m^2^ in the 5.85 GHz Wi-Fi band (origin country). However, these values are below the international reference levels: 10 W/m^2^ [54].

Additionally, when comparing the intensity levels recorded inside the airplane during the flight, the maximum values are inside the airplane with Wi-Fi service rather than without. However, the values are insignificant compared to the reference limits [54].

To our knowledge, no study has yet assessed individual exposure to RF-EMFs onboard an aircraft during flight, regardless of whether Wi-Fi service is available or not. The results were compared with studies and measurements conducted by the authors of this work in the Wi-Fi frequency bands across different microenvironments (Table 3).

This study was not easy, and as in other studies, it had some limitations. The main difficulty was obtaining information from the airline operating the flights. No technical issues occurred with the personal exposimeters, which were configured at the airport approximately 90 min before boarding to ensure sufficient battery life for the duration of the flight.

In addition, to transport the personal exposimeters, there was the need for an authorization certificate signed by the director of the research group at the University of Castilla-La Mancha. This certificate had to be presented to the airport police before passing through border control, but fortunately, no problems arose.

A common issue with all personal exposimeters is the recording and processing of nondetect data, which were identified in advance of the statistical analysis, as described in the methodology section.

As observed in the results, intensity levels changed from one flight to the other. In this study, the maximum values were recorded inside the airports (Madrid and CDMX) and when the airplane took off, for the first trip. The smallest values were recorded during the flight made with the airline that did not provide internet to its customers.

The maximum value recorded during both the outbound and return flights was influenced by the connection to and use of Wi-Fi by the researcher responsible for the measurements. This occurred at three different moments when the Wi-Fi service was contracted. As mentioned in Section 3, the use of the service had been planned in advance, including payment of the Wi-Fi fee to enable chatting on WhatsApp, searching for information on the internet, and receiving, downloading, and viewing a file containing information, pictures, and a short video. To monitor and synchronize events for this planned activity, a WhatsApp message was sent to a researcher in Spain, who responded with information during the flight, when connected to Wi-Fi and at the time of boarding.

The size of files and videos was small, and it was easy to download and view them. These events were recorded in a personal diary and corresponded with the peaks or maximum values shown in the graphs (for the outbound and the return flight, first trip). Considering these results, it can be deducted that installation and conditioning for Wi-Fi inside the aircraft responds to a connectivity request, if available; that is, it functions similarly to a normal on-demand router. For the use of a 2.4 GHz Wi-Fi band or 5.85 GHz Wi-Fi band, reception and transmission are assigned to different channels.

The other peaks observed on a smaller scale during the flight may correspond to Wi-Fi use by other passengers onboard. However, the results also show periods during the flight in which the intensity remained stable, without any high peaks. This stability was attributable to the absence of connectivity activity and Wi-Fi use, as passengers were asleep or inactive. The maximum values recorded during departure or arrival were the result of the airplane antennas connecting to ground antennas at the airport. This connection allowed the airplane to access a Wi-Fi network and communicate with the control tower.

We observed that the intensity levels of Radiofrequency Electromagnetic Fields were consistently lower during the return flight compared to the outbound flight (second trip, Madrid–Brussels), even though no Wi-Fi service was available onboard for passengers. This phenomenon has several possible technical explanations: (1) Internal emissions from the aircraft itself, as many aircraft are equipped with built-in Wi-Fi routers, even when the service is deactivated. These devices may still emit beacon signals on the Wi-Fi bands as part of the in-cabin system. (2) Passenger devices, as although passengers are advised to enable airplane mode, some may not do so correctly or may disable it to use Bluetooth. Mobile phones and tablets periodically emit probe requests searching for known networks, generating intermittent readings in the 2.4 or 5.85 GHz bands. (3) Cabin crew or in-flight entertainment system devices, which may include internal Wi-Fi modules that connect passenger tablets to a local server. These systems can remain active even if internet connectivity is unavailable. (4) Non-Wi-Fi sources of electromagnetic interference, such as onboard electronic equipment (proximity sensors, internal communications, microwave ovens, or LCD displays), which can emit spurious radiation in the ISM bands. (5) During ascent or descent, the exposimeter could detect emissions from Wi-Fi access points on the ground, especially below 3000 m altitude. These signals are difficult to isolate, making it challenging to determine the exact origin of the recorded intensity levels, representing a limitation of this study that could be addressed in future work.

The airplane is metallic, behaving like a Faraday cage; therefore, these exposure measurements in the Wi-Fi frequency band are not affected by other frequency bands, such as the band for mobile phones. For this reason, there is a need to set mobile phones to flight mode to connect to the Wi-Fi band [57] and contract the service.

This study is important because it provides experimental evidence on the levels of exposure to Radiofrequency Electromagnetic Fields in airplanes and airports, an increasingly connected environment. Its findings help protect public health, guide safety policies, and educate society about real versus perceived risks. This study is based on measurements carried out during two complete round trips (four flights in total). Although this represents a limited dataset that does not allow for general conclusions about EMF exposure in airplanes, it provides a proof-of-concept case study illustrating the feasibility and challenges of performing such measurements under real flight conditions.

## 5. Conclusions

The total average value recorded during the outbound flight was 93.9 µW/m^2^ with a 95th percentile of 336 µW/m^2^ in the 2.4 GHz Wi-Fi frequency band and 46.4 µW/m^2^ with a 95th percentile of 148 µW/m^2^ in the 5.85 GHz Wi-Fi frequency band (Spain to Mexico); a total average of 7.29 µW/m^2^ with a 95th percentile of 9.33 µW/m^2^ in the 2.4 GHz Wi-Fi band and 2.40 µW/m^2^ with a 95th percentile of 2.49 µW/m^2^ in the 5.85 GHz Wi-Fi band (Spain to Belgium).

The average values recorded during the return flight were 26.7 µW/m^2^ with a 95th percentile of 98.3 µW/m^2^ in the 2.4 GHz Wi-Fi frequency band and 9.87 µW/m^2^ with a 95th percentile of 38.7 µW/m^2^ in the 5.85 GHz Wi-Fi frequency band (Mexico to Spain); a total average of 3.24 µW/m^2^ with a 95th percentile of 6.10 µW/m^2^ in the 2.4 GHz Wi-Fi band and 1.23 µW/m^2^ with a 95th percentile of 1.51 µW/m^2^ in the 5.85 GHz Wi-Fi band (Belgium to Spain). All values are below the maximum allowed for personal exposure for the general public (10 W/m^2^) established by the applicable international guidelines.

The highest peaks or values were recorded inside the airport in the country of origin (Spain) and inside the airport in the destination country (Mexico), for the first two journeys, while the lowest value was recorded inside the airplane without internet service during the flight.

The results indicate that current smartphones receive and emit 5.85 GHz Wi-Fi signals with very low intensity. Personal exposure levels to RF-EMFs from the Wi-Fi frequency band, both inside an airplane and at the airport, are very low and far below reference levels established by international guidelines, for example, 336 µW/m^2^ equals 0.00336% of 10 W/m^2^ and 1500 µW/m^2^ equals 0.015%.

The results reported here should be interpreted as case-specific findings and not as generalized outcomes. Nevertheless, this exploratory work establishes a methodological baseline that can guide future studies. Additional measurement campaigns covering more flights, different aircraft types, routes, and conditions will be needed to compare and obtain a representative and generalized picture of EMF exposure in commercial aviation.

## Figures and Tables

**Table 1:** Total average personal exposure to RF-EMFs by microenvironments in two trips by airplane, from Madrid (Spain) to CDMX (Mexico), outbound and return flight. Intensity of electromagnetic wave in µW/m^2^ (all values are expressed with three significant figures).

Microenvironment	Outbound Flight		Microenvironment	Return Flight	
	2.4 GHz Wi-Fi (µW/m^2^)	5.85 GHz Wi-Fi (µW/m^2^)		2.4 GHz Wi-Fi (µW/m^2^)	5.85 GHz Wi-Fi (µW/m^2^)
Madrid airport	313	343	CDMX airport	451	110
inside the airplane	115	28.2	inside the airplane	160	46.6
taking off	319	305	taking off	126	12.9
**flying**	**93.9**	**46.4**	**flying**	**26.7**	**9.87**
landing	330	51.4	landing	76.2	17.6
inside the airplane	78.5	94.8	inside the airplane	45.2	23.6
CDMX airport	442	297	Madrid airport	445	96.6

**Table 2:** Total average personal exposure to RF-EMFs by microenvironments in two trips by airplane, from Madrid (Spain) to Brussels (Belgium), outbound and return flight. Intensity of electromagnetic wave in µW/m^2^ (all values are expressed with three significant figures).

Microenvironment	Outbound Flight		Microenvironment	Return Flight	
	2.4 GHz Wi-Fi (µW/m^2^)	5.85 GHz Wi-Fi (µW/m^2^)		2.4 GHz Wi-Fi (µW/m^2^)	5.85 GHz Wi-Fi (µW/m^2^)
inside the airplane (Madrid)	6.28	11.6	inside the airplane (Brussels)	7.73	8.50
taking off	10.2	2.39	taking off	6.87	3.32
**flying**	**7.29**	**2.40**	**flying**	**3.24**	**1.23**
landing	5.90	5.05	landing	3.19	2.38
inside the airplane (Brussels)	5.83	10.2	inside the airplane (Madrid)	4.85	9.28

**Table 3:** Minimum and maximum average values measured in some studies in the Wi-Fi frequency bands, in different microenvironments, and inside an airplane flying (the intensity of the electromagnetic wave in µW/m^2^).

Location and Publication	Intensity of Electromagnetic Wave in Some Microenvironments from Wi-Fi Band (in µW/m^2^)	
	Minimum	Maximum
Albacete (with volunteers) [14]	3.90	86.9
Albacete (school building: inside and outside) [24]	0.610	121
Jordan (University area) [23]	1.41	385
Mexico (with volunteers) [15]	93.3	500
Albacete (university building: inside a professor’s office and classroom with students) [22]	0.530	205
Cáceres (inside a house) [56]	0.0332	58.1
Inside the airplane flying (with internet service), this study	9.87	93.9
Inside the airplane flying (without internet service), this study	1.23	7.29

## Data Availability

The original contributions presented in this study are included in the article. Further inquiries can be directed to the corresponding author(s).
